# Bloch theorem dictated wave chaos in microcavity crystals

**DOI:** 10.1038/s41377-023-01156-9

**Published:** 2023-05-04

**Authors:** Chang-Hwan Yi, Hee Chul Park, Moon Jip Park

**Affiliations:** 1 Center for Theoretical Physics of Complex Systems, Institute for Basic Science (IBS), Daejeon, 34126 Republic of Korea; 2 Department of Physics, Pukyong National University, Busan, 48513 Republic of Korea; 3 Department of Physics, Hanyang University, Seoul, 04763 Republic of Korea

**Keywords:** Micro-optics, Photonic devices

## Abstract

Universality class of wave chaos emerges in many areas of science, such as molecular dynamics, optics, and network theory. In this work, we generalize the wave chaos theory to cavity lattice systems by discovering the intrinsic coupling of the crystal momentum to the internal cavity dynamics. The *cavity-momentum locking* substitutes the role of the deformed boundary shape in the ordinary single microcavity problem, providing a new platform for the in situ study of microcavity light dynamics. The transmutation of wave chaos in periodic lattices leads to a phase space reconfiguration that induces a dynamical localization transition. The degenerate scar-mode spinors hybridize and non-trivially localize around regular islands in phase space. In addition, we find that the momentum coupling becomes maximal at the Brillouin zone boundary, so the intercavity chaotic modes coupling and wave confinement are significantly altered. Our work pioneers the study of intertwining wave chaos in periodic systems and provide useful applications in light dynamics control.

## Abstract (web summary)

We discover wave chaos in a photonic crystal dictated by the Bloch vector by demonstrating its interrelation with geometric morphology-originated wave chaos in single microcavities.

## Graphical abstract

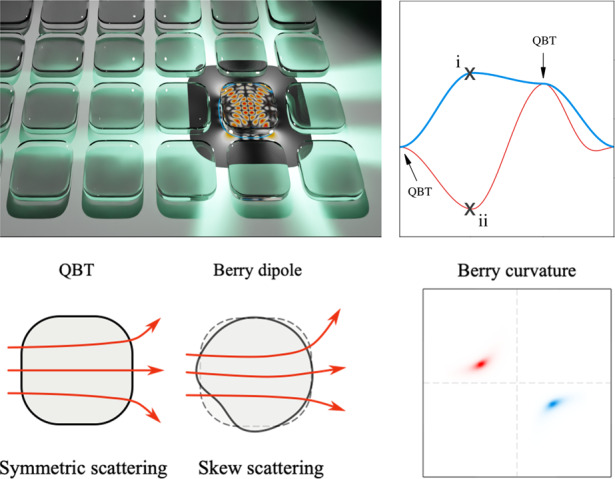

## Introduction

Light dynamics in optical microcavity^1–6^ provides the prominent platform to study quantum-classical correspondence, formally known as the field of quantum chaos^7–9^. Understanding the chaotic signatures in this transitional regime promotes future technological applications^10–13^ emerging at the interface between classical and quantum mechanics realms or, equivalently, ray and wave realms in optical microcavities^1,2,14–16^. Yet, in a more general sense, the level statistics of the microcavity mirror universal behaviors observed in various chaotic physical systems, such as Rydberg atoms^17–19^, ultra-cold atoms^20–22^, quantum dots^23–25^, and many-body systems^26–29^.

The boundary deformation (BD) in microcavity has been considered the most common approach for phase space engineering to yield the desired optical properties. The main idea is based on the observation that BD sensitively reconfigures the underlying phase space transportation^30,31^. Alternative approaches also have been proposed, such as tailoring phase space by a defect^32^ or including a circular hole inside the cavity^33,34^. Despite these comprehensive efforts, previous approaches still require a holistic device change at the fabrication stage, which remains the main obstacle to further rapid progress. In this regard, developing a new platform on which light dynamics can be studied by in situ experimental control is genuinely appealing. Here, we propose a two-dimensional periodic lattice structure consisting of multiple chaotic microcavities as a promising breakthrough. We reveal that the external crystal momentum coupling to the internal cavity dynamics can take over the role of the BD by breaking and restoring the inherent symmetry of the cavity. Both the direction and the amplitude of the external momentum can be readily controlled by steering the coherent excitation light sources. As a result, cavity lattice systems promise a hold for feasible control of wave chaos features such as dynamical localization and tunneling^35–39^.

In this work, we explore chaotic signatures in periodic lattices dictated by the Bloch theorem for the first time. We study the adiabatic change of the internal cavity states due to the crystal momentum coupling as

1 $${\Psi}_{{{{\mathrm{tot}}}}}\left( {{{\mathbf{r}}}} \right) = e^{{{{\mathrm{i}}}}{{{\mathbf{k}}}}\cdot {{{\mathbf{r}}}}}\psi _{{{{\mathrm{int}}}}}\left( {{{\mathbf{r}}}} \right)$$

where ${\Psi}_{{{{\mathrm{tot}}}}}\left( {{{\mathbf{r}}}} \right)$ and **k** represent the total wave function over the lattice and the crystalline momentum, respectively. Here, $\psi _{{{{\mathrm{int}}}}}\left( {{{\mathbf{r}}}} \right)$ describes the internal dynamics of the cavity states, which we focus on. The coupling between the internal phase space dynamics and the crystal momentum is observed explicitly. In particular, the momentum-induced coupling leads to the hybridizations of the wave function from the scar states to the regular orbit states; namely, we observe the crystal momentum-induced *dynamical localization transition* of the internal dynamics. On the lattice scale, we find that introducing the additional deformation leads to the finite Berry curvature and Hall effect triggered by skew scattering events. Our work firstly promotes applications to topological optical transport utilizing chaotic states. Finally, we discuss the possibility of extending our studies to various lattice systems.

**Fig. 1:**
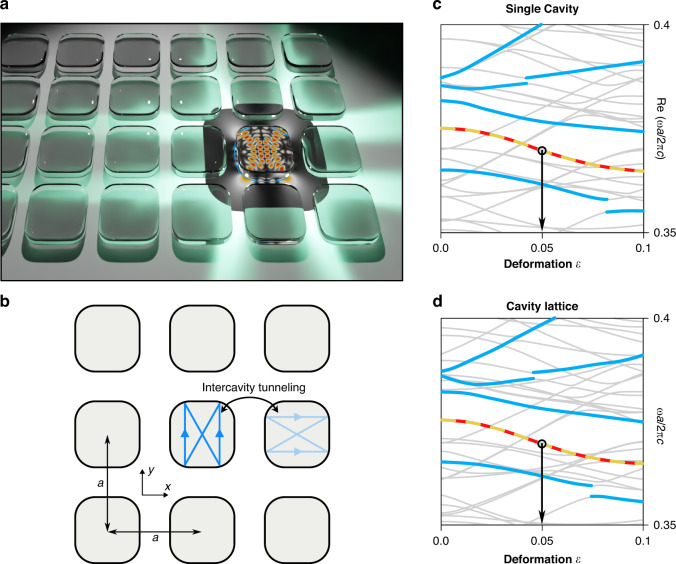
Bloch theorem dictated scar lattice. **a** Conceptual illustration of a photonic crystal consisting of a deformed dielectric microcavities. **b** Schematic diagram of a square lattice unit-cell with a lattice constant *a*. **c** Real part resonant frequencies in a single cavity as a function of the cavity deformation *ε*. **d** Energy eigenvalues in a cavity array lattice as a function of *ε* at zero crystalline momentum **k** = 0 [Γ in Fig. 4a]. In **c** and **d**, successive Demkov-type couplings^56^ for the stable regular modes and the scar modes are highlighted by thick curves. The arrows at *ε* = 0.05 mark the degenerated scar modes (solid and dashed curves) we study

We consider a square lattice consisting of a single dielectric cavity with a relative refractive index, $n_{{{{\mathrm{in}}}}}/n_{{{{\mathrm{out}}}}} = 10$, inside the cavity [see Fig. 1]. With this high refractive index, we can find energetically well-isolated target modes by suppressing undesired overlaps with other extra modes. However, our result is generally applicable to the broad range of the refractive index. The boundary of each cavity is determined by the four-fold rotational symmetric (C_4_) deformation, which can be represented as, $r\left( {\theta ;\varepsilon } \right) = r_0\left[ {1 - \varepsilon \cos \left( {4\theta } \right)} \right]$, where *ε* denotes the deformation strength. $r_0 = R/\sqrt {1 + \varepsilon ^2/2}$, where *R* is the radius of the circle when *ε* = 0, is the normalization constant preserving a cavity area under the variation of *ε*. The Helemholtz equation, $- \nabla ^2\vec \psi = n^2\left( {x,y} \right){\textstyle{{\omega ^2} \over {c^2}}}\vec \psi$, is solved to obtain resonant frequencies *ω* of the transverse-magnetic (TM)-polarized modes $\left[ {\vec \psi = \left( {0,0,E_z} \right)} \right]$, where *c* is a speed of light. For TM modes, the waves and their normal derivative are set to be continuous across the cavity boundary. The two lattice constants, where we impose a periodic boundary condition accordingly, are given by |*a*_*x*_| = |*a*_*y*_| = *a* = 2.2*R*. To numerically obtain the modes both in a single cavity and a lattice, we implement the boundary element method^40–43^.

**Fig. 2:**
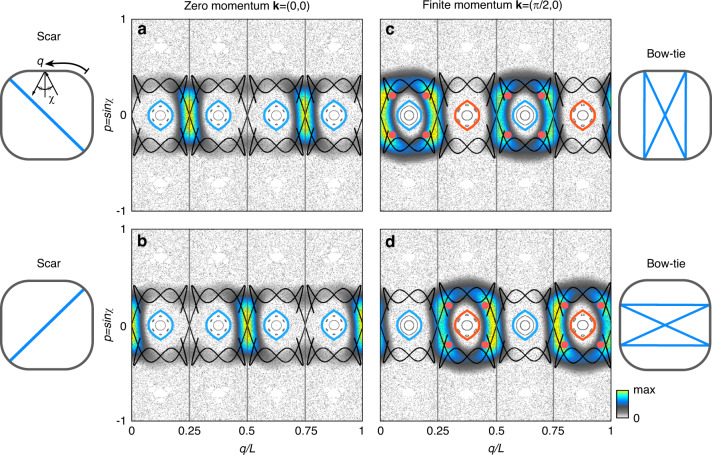
Phase space of deformed cavity. Husimi functions for modes in a cavity lattice superimposed on the ray-dynamical classical phase space (underlying gray dots) in Birkhoff-coordinate $(q/L,p = \sin \chi )$, where *q*/*L* is arclength normalized by a cavity perimeter *L* and *χ* an incident angle of ray. In (**a**)–(**d**), the four large closed islands and the crossing points of stable and unstable manifolds (black curves) at *p* ~ 0 correspond to period-2 stable and unstable orbits, respectively. The orange dots in (**c**) and (**d**) represent the phase space points of the bow-tie orbit. **a**, **b** The Husimi distributions for the scar states obtained when ${{{\mathbf{k}}}} = \left( {k_{{{\mathrm{x}}}},k_{{{\mathrm{y}}}}} \right) = \left( {0,0} \right)$, while (**c**) and (**d**) are for the bow-tie orbit states induced by couplings of the two scar states when ${{{\mathbf{k}}}} = \left( {\pi /2,0} \right)$. The leftmost and rightmost panels show corresponding classical orbits in real space. The four vertical lines in (**a**–**d**) indicate a quarter arclength interval *L*/4. When **k** = (0, 0) (**a**, **b**), the phase space (e.g., islands and manifolds) exhibits an *L*/4 translation symmetry, and the Husimi functions in (**a**) and (**b**) are distributed mutually exclusively. On the other hand, when **k** = (*π*/2, 0) (**c**, **d**), the translation symmetry of the phase space breaks, and the Husimi distributions in (**c**) and (**d**) have large overlaps around the vertical lines. Detailed correspondences between the Bloch momenta **k** and the phase space deformation can be found in Supplementary Materials. The continuous evolution of the Husimi function and the underlying phase space over the range [*π*/4 ≦ arc tan(*k*_*y*_/*k*_*x*_) ≦ 9*π*/4] for |**k**| = *π*/2 is shown in the supplementary animations: “Ani_supple_phc_hus1_series.mov” and “Ani_supple_phc_hus2_series.mov”

Under a weak BD, invariant tori in the ray-dynamical phase space of the integrable system are destroyed according to the Kolmogorov-Arnold-Moser (KAM) and Poincaré-Birkhoff theorems^44,45^. As the deformation increases, the regular and chaotic regions fill the phase space before it becomes fully chaotic and ergodic. Figure 2 shows the phase space in the Birkhoff-coordinate $(q/L,p = \sin \chi ) \in \left[ {0,1} \right] \times \left[ { - 1,1} \right]$^46^. Here, *q/L* is the normalized boundary arclength where the ray bounces off (*L* is a cavity perimeter), and *χ* is the incident angle of the ray. The mixed phase space in the figure shows the island structures of regular motions surrounded by a chaotic sea. See Supplementary Materials for details.

## Results

Two degenerate scar modes^47^, the non-trivial localization on the unstable fixed points, are observed in the single cavity case for *ωR/*2*πc* ≈ 0.375 at *ε* = 0.05. A series of thick blue curves in Fig. 1c show the successive regular and scar modes in which a similar sequence has been analyzed^48^. The scar modes form a pair under the underlying C_4_-symmetry. In the lattice, with the identical cavity geometry, the spectra at zero momentum exhibit very similar energy eigenstates (Fig. 1d). We observe the pair of scar modes equally for almost the same energy as the single cavity case. Figure 2a, b shows the Husimi distributions of the two degenerate scar states, which are localized on top of the distinct unstable fixed points. These fixed points correspond to period-2 (i.e., two bounces for one cycle of a classical orbit) unstable periodic orbits shown in the leftmost panels in Fig. 2. The black curves in Fig. 2 show the stable and unstable manifolds of the period-2 unstable orbit, and the unstable fixed points corresponding to this orbit are the crossing points of those manifolds. Note that the Husimi distributions are obtained for the cavity boundary wave, and the inside-incident version was employed^49,50^.

We now consider the inclusion of the finite crystal momentum ${{{\mathbf{k}}}} = \left( {k_x,k_y} \right)$. In the presence of the non-zero crystal momentum, we observe the degeneracy lifting of the two scar modes. This lifting indicates the state hybridization due to the C_4_-rotational symmetry breaking of the internal wave function with the non-zero momentum. The hybridized states are found to localize prominently around the period-4 bow-tie orbit (see the rightmost panels in Fig. 2), the satellite orbit of the central period-2 island. Figure 2c, d exemplifies the Husimi distributions for these hybridized modes localized on the bow-tie orbits. The mode evolution depending on the crystalline momentum variations in cavity lattices can be equivalently realized in single cavities by applying the additional deformation perturbation. See Supplementary Materials for detailed demonstrations.

**Fig. 3:**
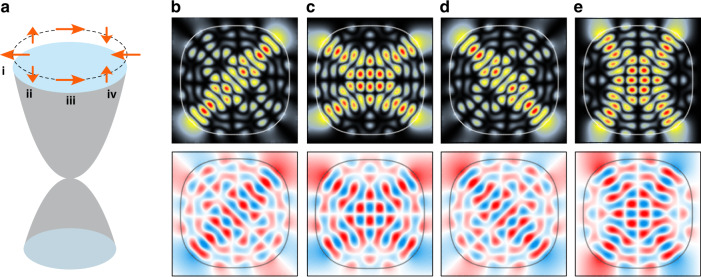
Coupling between the internal phase space dynamics and the crystal momentum. **a** Schematic illustration of the pseudo-spin evolution along the encircling around the zero crystalline momentum $(k_x,k_y) = \left( {0,0} \right)$ (Γ in Fig. 4a). **b**–**e** The upper and lower panels display the square modulus |*ψ*|^2^ and the real part Re(*ψ*) of wave functions corresponding to, respectively, i–iv in (**a**). The directions of the crystalline momentum for (**b**), (**c**), (**d**), and (**e**) are equivalent to the ones of $(k_x,k_y) = \left( {\pi , - \pi } \right),\left( {\pi ,0} \right),\left( {\pi ,\pi } \right),{\rm{and}}\left(0,\pi\right)$, respectively

**Fig. 4:**
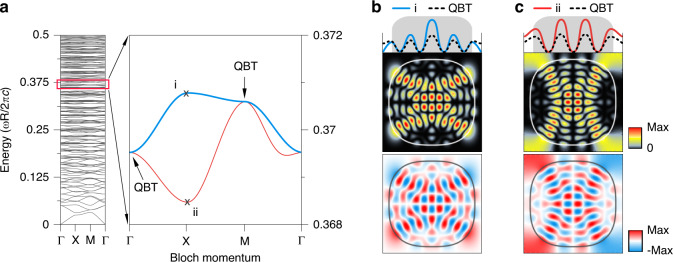
Band structures and scar modes. **a** Energy bands as a function of the crystalline momentum $(k_x,k_y)$ for a fixed cavity deformation *ε* = 0.05. The zoomed bands correspond to the ones indicated by the arrow in Fig. 1d. **b** Spatial distribution of the even parity mode $\left( {\psi _{{{\mathrm{E}}}}} \right)$ in a unit-cell for $(k_x,k_y) = \left( {\pi ,0} \right)$ (*i* in **a**). The upper panel depicts the wave projection onto the *x*-axis showing the vanished wave at the unit-cell boundary and the dense confinement of the wave inside the cavity region (shaded rectangle). **c** The same as (**b**) but for the odd parity mode (*ψ*_O_) (*ii* in **a**). The upper panel depicts the intense wave distribution outside the cavity, including the unit-cell boundary

In addition, Fig. 3b–e shows the hybridized states that have strong directional anisotropy in the direction of the crystal momentum. To be specific, the hybridization of the two scar modes are numerically obtained and described by the effective Hamiltonian, given as^51^,

2 $$H_{{{{\mathrm{SO}}}}} = \frac{{{\Delta}_{{{{\mathrm{hyb}}}}}}}{2}\left( {\cos k_x - \cos k_y} \right)\sigma _x + {{{\mathrm{V}}}}_0\sin k_x\sin k_y\sigma _z$$

where *σ* represent the Pauli matrices for the scar state degree of freedom. ∆_hyb_ = 1.156 × 10^−3^ represents the hybridization strength between the two scar modes, while V_0_ = 0.213 × 10^−3^ represents the relative energy shift of the two states, respectively. Since the two scar states are energetically well-decoupled from other states, we can consider the two scar states as the effective spinor (|↑〉 and |↓〉). Under this spinor representation, the spinor wind twice around the loop encircling the zero momentum (Fig. 3a). This winding of the spinor gives rise to the 2*π*-Berry phase: $\gamma \equiv i{\oint} \left\langle \psi \left( {{{\mathbf{k}}}} \right)\left| {\nabla _{{{\mathbf{k}}}}} \right|\psi \left( {{{\mathbf{k}}}} \right)\right\rangle d{{{\mathbf{k}}}} = 2\pi$, where |*ψ*(**k**)〉 indicates one of the scar states and $i = \sqrt { - 1}$. The degeneracy of the spinor scar states manifests as the topological quadratic band touchings (QBT) protected by C_4_-symmetry in the momentum space (see Fig. 4a)^52^. Here, the mode hybridization due to the crystalline momentum is not restricted in the scar modes and is valid for the general modes that exhibit a degeneracy at time-reversal invariant momenta.

### Maximal momentum coupling

When the momentum becomes maximal near the Brillouin zone (BZ) boundary [X = (*π*,0) and Y = (0*,π*)], the wave function characteristics in the whole unit-cell domain deviate significantly due to the change in the boundary condition. For instance, at X point, the internal wave function satisfies the anti-periodic boundary condition on the unit-cell boundary: *ψ*_Int_(*x* + *a, y*) = −*ψ*_Int_(*x, y*) and *ψ*_Int_(*x,y* + *a*) = *ψ*_Int_(*x, y*). The linear combination of the two scar states forms two regular states depending on its parity, [*ψ*(*x,y*) = ±*ψ*(−*x,y*)], due to C_4_-rotational symmetry. At the X point in BZ, there exist only two possibilities that the resonant energy of the regular state can adiabatically deform. First, even parity regular state, *ψ*_E_(*x,y*) (Fig. 4b), is incompatible with the anti-periodic boundary condition unless the wave function vanishes at the unit-cell boundary. The vanishing wave function manifests as the flat band with zero group velocity (see the upper band along the X-M interval in Fig. 4a). In addition since *ψ*_E_(*x,y*) is confined more densely inside the cavity, where the refractive index is higher, the effective wavelength is reduced, i.e., even parity states form higher energy states (point “i” in Fig. 4a).

On the other hand, the odd parity regular state, *ψ*_O_(*x,y*) (Fig. 4c), shows the compensating intensity enhancement at the unit-cell boundary since the even and odd wave functions must form the complete basis of the two original scar modes. Hence, it results in lengthened effective wavelength (i.e., more waves are outside the cavity, the lower refractive index region) and manifests as the lower energy resonant states (point “ii” in Fig. 4a). Finally, when the momentum vector reaches the BZ corner [M = (*π,π*)], the global C_4_-rotational symmetry is restored. In this case, the hybridization disappears to form the degenerate QBT again, which can be interpreted as a revived dynamical localization of the spinor scar modes.

### Skew boundary scattering in Chaotic states

**Fig. 5:**
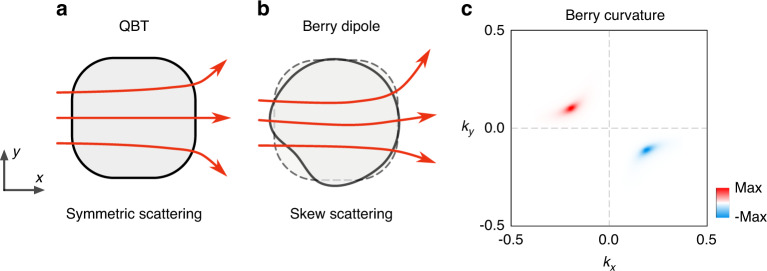
Skew scattering due to the Berry curvature. Schematic illustration of BDs. **a** Original C_4_-symmetric boundary with QBT and **b** C_4_-breaking boundary. C_4_-breaking boundary perturbation splits the single QBT into a pair of Dirac cones. Subsequently, C_2_-breaking boundary perturbation gaps out the Dirac cones and induces the Berry curvature dipole. The Berry curvature dipole induces the skew scattering depending on the incident momentum, *k*_inc_. **c** Distribution of non-zero Berry curvature in the momentum space induced by scar states

At last, we consider the scar states in the presence of the generic BD perturbation, which is given as *δr*(*θ*) = *ϵ*_1_ cos(*Nθ* + *ϕ*_0_). For the even-number oscillating BD [*N* ∈ 2Z] [e.g., (*N,ϵ*_1_*,ϕ*_0_) = (2,0.01,0)], which preserves C_2_ symmetry, the rotational symmetry of the cavity is lowered from C_4_ to C_2_ symmetry, the topological protection of QBT is lost. Correspondingly, the QBT-carrying 2*π*-Berry phase can split into a couple of the Dirac linear band crossings, which of each is associated with the *π*-Berry phase. On the other hand, for odd-number oscillating BD [*N* ∈ 2Z + 1], C_2_-symmetry breaking induces the skew-symmetric scattering of the chaotic modes [see Fig. 5b], which induces the non-zero Berry curvature $\left\{ {{\Omega}_{xy}\left( {{{\mathbf{k}}}} \right) = i\left[ {\left\langle\nabla _{k_x}\psi \left( {{{\mathbf{k}}}} \right)\vert \nabla _{k_y}\psi \left( {{{\mathbf{k}}}} \right)\right\rangle - \left( {y \leftrightarrow x} \right)} \right]} \right\}$ in the momentum space. Figure 5c shows the calculated Berry curvature for $\left[ {\left( {N,{\it{\epsilon }}_1,\phi _0} \right) = \left( {5,0.01,{\textstyle{\pi \over {10}}}} \right)} \right]$. Since the underlying time-reversal symmetry ensures the antisymmetry of the local Berry curvature $\left[ {{\Omega}_{xy}\left( {{{\mathbf{k}}}} \right) = - {\Omega}_{xy}\left( { - {{{\mathbf{k}}}}} \right)} \right]$, the total sum (monopole) of the curvature vanishes. Accordingly, the skew scattering by the BD is characterized by the finite dipole moment of the local Berry curvature^53,54^:

3 $$D_j = \mathop {\sum}\limits_{{{\mathbf{k}}}} {\partial _{k_j}{\Omega}_{xy}\left( {{{\mathbf{k}}}} \right)}$$

where *j* = *x,y*.

Since the optical microcavity array can be excited by the coherent beam source with a well-defined incident momentum, the Berry curvature dipole can be measured through the skew-symmetric beam transport. The semi-classical transport equation^55^ describes the light dynamics of the Mie regime, and the non-zero Berry dipole manifests as the effective magnetic field as,

4 $${{{\mathbf{v}}}}_{{{\mathbf{k}}}} = \frac{{\partial E_{{{\mathbf{k}}}}}}{{\partial {{{\mathbf{k}}}}}} + {{{\dot{\mathbf k}}}} \times {\Omega}_{xy}\left( {{{\mathbf{k}}}} \right)\hat z,\;{{{\dot{\mathbf k}}}} = \nabla n\left( {{{\mathbf{r}}}} \right)$$

where **v**_**k**_ is the group velocity vector of the scar states having energy *E*_**k**_, and *n*(**r**) is the slowly varying local refractive index. The second term $\left( {{{{\dot{\mathbf k}}}} \times {\Omega}_{xy}} \right)$ in Eq. (4) gives rise to skew-symmetric light transport that can be captured by control of the incident beam momentum **k**. The explicit demonstration of the skew-symmetric light transportation can be found in Supplementary Materials.

## Discussion

To conclude, we have studied the wave chaos of the deformed cavity coupled to the external crystalline momentum in a periodic cavity array. The external crystalline momentum now replaces the role of the boundary shape deformation. By controlling the momentum, we observe the dynamical localization transitions. Our work provides a new promising platform, enabling the in situ study of various wave chaos phenomena. For example, if the additional higher energy and lower energy states are involved, it can induce crystal momentum-induced dynamical tunneling phenomena. This will be the topic of future study.

In addition, we note that, contrary to the previous studies of the topological photonic crystals (which mainly focus on the Rayleigh regime), the governing dynamics of the chaotic state in the Mie regime is described by the semi-classical transport. In this work, we propose, for the first time, the possibility of realizing Berry curvature-induced transport phenomena that utilize the intrinsic wave property of the chaotic states. The crossover between Rayleigh and Mie regime of Berry curvature-induced transport would pioneer a new aspect of wave-particle correspondence in the field of wave chaos.

Our work can be generalized in a few different aspects. Though we have considered the simple square lattice, many other two-dimensional lattices are expected to show qualitatively different behaviors. For instance, away from a simple Bravais lattice, the deformed cavity in Lieb and Kagome lattice is expected to produce a flat band. The intrinsic localization properties would give rise to a stronger coupling within a unit-cell. Further studies on the various lattice systems and wave chaos would also be an intriguing topic for future study.

## Methods

We solve the Helmholtz wave equation, which is deduced from Maxwell’s equations without sources, for optical modes in single cavities and photonic crystals,

5 $$- \nabla ^2\vec \psi = n^2\left( {{{\mathbf{r}}}} \right)\frac{{\omega ^2}}{{c^2}}\vec \psi$$

where *n*(**r**) and *ω* = *ck* are, respectively, the refractive index of the piecewise homogeneous medium and the free-space temporal frequency with vacuum wavenumber *k* and speed of light *c*. Given the cavity boundary shape, *R*(*θ*;*ε*) = *R*_0_[1 − *ε*cos(4*θ*)], where $R_0 = \rho /\sqrt {1 + \varepsilon ^2/2}$, we set *n*(**r**) = 10 for |**r**| *< R*(*θ*;*ε*) and *n*(**r**) = 1 otherwise. *ρ* is the radius of a circle when *ε* = 0. The lattice constant is given as |*a*_*x*_| = |*a*_*y*_| = *a* = 2.2*ρ*. We focus on the transverse magnetic [TM; $\vec \psi = \left( {0,0,E_z} \right)$] polarization of modes. The TM-polarized modes fulfill the dielectric boundary condition that *E*_*z*_ and are continuous across the boundary interface of two different refractive index domains. Here, $\vec \nu$ is an outward normal vector of the boundary. Note that the transverse-electric [TE; $\vec \psi = \left( {0,0,H_z} \right)$] modes satisfy a different boundary condition: *H*_*z*_ and are continuous, yet, still the solution of the same wave equation, Eq. (5). In addition to the dielectric boundary conditions, we impose a two-dimensional periodic condition at the unit cell boundary of the two-dimensional periodic lattice. A pure outgoing wave condition at infinity is applied for the modes in the single cavities. To solve Eq. (5) numerically, we employ the boundary element method (BEM)^40,41^ and further implement the block Sakurai–Sugiura method^42,43^ to compute the optical modes more efficiently.

## Supplementary information

Supplementary Materials for “Bloch Theorem Dictated Wave Chaos in Microcavity Crystals”

Bloch momentum-dependent mode evolution 1

Bloch momentum-dependent mode evolution 2

Deformation-dependent mode evolution 1

Deformation-dependent mode evolution 2

Bloch momentum-dependent Husimi function evolution 1

Bloch momentum-dependent Husimi function evolution 2

Deformation-dependent phase space evolution
