# Supplementary material for: Bloch theorem dictated wave chaos in microcavity crystals
Source: Light Sci Appl. 2023 May 4;12:106. doi: 10.1038/s41377-023-01156-9 (PMC10160058; doi:10.1038/s41377-023-01156-9)
Supplement: Supplementary file 1 — Supplementary Materials for “Bloch Theorem Dictated Wave Chaos in Microcavity Crystals” [file 41377_2023_1156_MOESM1_ESM.pdf]

Supplementary Information for

"Bloch Theorem Dictated Wave Chaos in Microcavity Crystals"

Chang-Hwan Yi[1], Hee Chul Park[1,2,a†], Moon Jip Park[1,3,b†]

[1]Center for Theoretical Physics of Complex Systems, Institute for Basic Science (IBS), Daejeon, 34126, Republic of Korea

[2]Department of Physics, Pukyong National University, Busan 48513, Republic of Korea

[3]Department of Physics, Hanyang University, Seoul 04763, Republic of Korea

E-mail: [a]hc2725@gmail.com, [b]moonjippark@hanyang.ac.kr

[†]Corresponding authors equally contributing to this work.

Contents

Page

S1 Influence of the Bloch wavevector on the wave propagators in the periodic structures 3

S1.1 Path integral with Bloch momenta and Jacobi θ-function 3

S1.1.1 Eigenstates of the Helmoholtz operator in periodic systems 3

S1.1.2 Construction of a propagator with Jacobi θ-function 4

S1.1.3 Euler-Maclaurin formula 4

S1.1.4 Closed form of propagator embodying Bloch momenta effects 6

S2 Effective equivalence between boundary deformation and Bloch momenta 7

S2.1 Two-band Hamiltonian of QBT for cavity lattices and its interpretation in terms of a single cavity deformation 7
S2.2 Realization of the boundary deformation in a single cavity corresponding to the Bloch momentum in cavity lattices 8
S2.2.1 Basis shape function of the boundary deformation perturbation 9
S2.2.2 Bloch momentum direction and strength of the boundary deformation perturbation 9
S2.2.3 Equivalence of the energy dispersion due to the Bloch momenta and the boundary deformation 11
S2.2.4 Equivalence of the wavfunction evolution for the Bloch momenta and the doundary deformation 12
S2.2.5 Poincaré section of phase space 12

S3 Skew scattering light transportation 16

S1 Influence of the Bloch wavevector on the wave propagators in the periodic structures

In Sections S1 and S2, we show that the inclusion of the Bloch momenta in the cavity lattice systems can be effectively interpreted as the additional deformation in the single cavity systems. To this end, we begin with the path integral description of the wave propagator in the periodic space embodying the effects of the Bloch momenta in the reconfigured action.

S1.1 Path integral with Bloch momenta and Jacobi θ-function

S1.1.1 Eigenstates of the Helmoholtz operator in periodic systems

Let's consider the 1-D Helmholtz equation describing a dynamics in a unit-circular ring domain. The ring has a perimeter $2\pi R$, i.e., $\chi \in [0, 2\pi R)$ and is composed of homogeneous medium with a refractive index $\mathcal{N}$. Assuming time harmonic fields $\Psi(\chi, t) = \psi(\chi)e^{-i\omega\tau}$, we have

$$\frac{1}{\mathcal{N}^2}\nabla_\chi^2\Psi = \frac{1}{c^2}\frac{\partial^2\Psi}{\partial\tau^2} = \frac{\omega^2}{c^2}\psi \iff H\psi = -\frac{1}{\beta^2\mathcal{N}^2}\nabla_x^2\psi = \frac{i}{\beta}\frac{\partial}{\partial t}\psi = E\psi \tag{S1}$$

where $\chi = \beta x\frac{c}{\omega}$ and t is effective time with spatial dimension. Under the periodic boundary condition, $\psi(0) = \psi(L = \frac{2\pi R\omega}{\beta c})$, the eigenstates, $\psi_n(x)$, and the eigenvalues, E_n, are given as follows:

$$\psi_n(x) = \langle x|n\rangle = \frac{1}{\sqrt{L}}e^{2\pi i\frac{nx}{L}}\ ,\ E_n = \left(\frac{2\pi n}{\beta\mathcal{N}L}\right)^2\ , \quad n \in \mathbb{Z} \tag{S2}$$

Now, we consider a finite boundary flux, λ (playing a role like a momentum), characterized by the boundary condition, $\psi(L) = e^{2\pi i\lambda}\psi(0)$, so that the eigenstates and the eigenvalues are modified as follows:

$$\psi_n(x) = \langle x|n\rangle = \frac{1}{\sqrt{L}}e^{2\pi i\frac{(n+\lambda)x}{L}}\ ,\ E_n = \left(\frac{2\pi(n+\lambda)}{\beta\mathcal{N}L}\right)^2\ , \quad n \in \mathbb{Z} \tag{S3}$$

We simplify the eigenstate expression as

$$E_n = \upsilon\pi(n+\lambda)^2\ ,\ \upsilon \equiv \frac{4\pi}{(\beta\mathcal{N}L)^2} \tag{S4}$$

S1.1.2 Construction of a propagator with Jacobi θ-function

Having computed the eigenstates and the eigenvalues, we can now derive the propagator, $U(x,t)$ (in unit of E, i.e., in terms of the equaion of motion $H\psi = \frac{i}{\beta}\partial_t \psi$), as follows:

$$\begin{aligned} U(x,t) \equiv \langle x| e^{-i\beta H t} |0\rangle &= \sum_{n,n' \in \mathbb{Z}} \langle x | n \rangle \langle n | e^{-iHt} | n' \rangle \langle n' | 0 \rangle && \text{(S5)} \\ &= \sum_{n\in\mathbb{Z}} e^{-i\beta E_n t} \langle x | n \rangle \langle n | 0 \rangle && \text{(S6)} \\ &= \frac{1}{L} \sum_{n\in\mathbb{Z}} e^{-i\beta\pi v t (n+\lambda)^2} e^{2\pi i \frac{(n+\lambda)x}{L}} && \text{(S7)} \end{aligned}$$

The obtained propagator can be expressed by the Jacobi θ-function, as follows:

$$\begin{aligned} U(x,t) &= \frac{1}{L} \sum_{n=-\infty}^{\infty} e^{-i\beta\pi v t (n^2 + 2\lambda n + \lambda^2)} e^{2\pi i \frac{x}{L} n} e^{2\pi i \frac{x}{L}\lambda} && \text{(S8)} \\ &= \frac{1}{L} \sum_{n=-\infty}^{\infty} e^{-i\beta\pi v t n^2} e^{2\pi i \left(\frac{x}{L} - \beta v t \lambda\right) n} e^{2\pi i \left(\frac{x}{L}\lambda - \beta \frac{v t \lambda^2}{2}\right)} && \text{(S9)} \\ &= \frac{1}{L} \theta(z,\tau) e^{2\pi i \left(\frac{x}{L}\lambda - \beta \frac{v t \lambda^2}{2}\right)} && \text{(S10)} \end{aligned}$$

where $z = \left(\frac{x}{L} - \beta v t \lambda\right)$ and $\tau = -\beta v t$. The last line in the above follows the formal expression of the Jacobi θ-function, given as follows:

$$\theta(z,\tau) = \sum_{n=-\infty}^{\infty} e^{i\pi\tau n^2 + 2\pi i z n} = 1 + 2\sum_{n=1}^{\infty} e^{i\pi\tau n^2} \cos(2\pi z n) \quad \text{(S11)}$$

We introduce an additional regulator $\eta \to 0^+$ into the exponent,

$$\theta(z,\tau) = \lim_{\eta\to 0^+} \left[1 + 2\sum_{n=1}^{\infty} e^{-(\eta - i\pi\tau)n^2} \cos(2\pi z n) \right] \quad \text{(S12)}$$

in order to approximate the discrete summation to the integral by exploiting the Euler-Maclaurin formula for $f(x) = e^{-(\eta - i\pi\tau)x^2} \cos(2\pi z x)$.

S1.1.3 Euler-Maclaurin formula

If m and n are natural numbers, and $f(x)$ is a real or complex-valued continuous function for real numbers x in the interval $[m,n]$, then the integral, $I = \int_m^n f(x)dx$, can be approximated by

the sum (or vice versa), $S = f(m+1) + \cdots + f(n-1) + f(n)$, through the rectangle method. The Euler-Maclaurin formula provides for the difference between the sum and the integral in terms of the higher derivatives $f^{(k)}(x) = \frac{d^k}{dx^k} f(x)$ at the two endpoints of the interval, i.e., at $x = m$ and $x = n$. Thereby, it explicitly reads,

$$S = I + \sum_{k=1}^{p} \frac{B_k}{k!} \left[f^{(k-1)}(n) - f^{(k-1)}(m) \right] + R_p \tag{S13}$$

where B_k is the kth Bernoulli number (with $B_1 = \frac{1}{2}$), and R_p is an error term which depends on n, m, p, and f and is usually small for suitable values of p.

$$\begin{aligned} \theta(z,\tau) \approx 1 + 2 \lim_{\eta \to 0^+} \left[\int_0^\infty e^{-(\eta - i\pi\tau)x^2} \cos(2\pi z x) dx \right] \\ + 2 \lim_{\eta \to 0^+} \left[\sum_{k=1}^{\infty} \frac{B_k}{k!} \left[f^{(k-1)}(\infty) - f^{(k-1)}(0) \right] + R_p \right] \end{aligned} \tag{S14}$$

As the regulator goes to zero, $\eta \to 0^+$, the contribution of the asymptotic expansion in the second bracket vanishes. It is because, all the terms in the second bracket are expressed with a finite value or positive order in η when $\eta \to 0^+$, while the integral in the first bracket diverges.

Now by exploiting the standard Gaussian integral formulae,

$$\int_{-\infty}^{\infty} e^{-ax^2} e^{bx} e^{c} dx = \sqrt{\frac{\pi}{a}} e^{\frac{b^2}{4a} + c} \tag{S15}$$

and $1 + 2 \int_0^\infty e^{-\xi x^2} \cos(\sigma x) = \int_{-\infty}^{\infty} e^{-\xi x^2} e^{i\sigma x}$, we can obtain the asymptotic expression of θ-function, as follows:

$$\theta(z,\tau) \sim \lim_{\eta \to 0^+} \sqrt{\frac{\pi}{\eta - i\pi\tau}} e^{\frac{\pi^2 z^2}{\eta - i\pi\tau}} \tag{S16}$$

S1.1.4 Closed form of propagator embodying Bloch momenta effects

Plugging Eq. (S16) into Eq. (S10), with $z = \left(\frac{x}{L} - \beta v t \lambda\right)$, $\tau = -\beta v t$, and $v = \frac{4\pi}{(\beta \mathcal{N} L)^2}$, we can yield the final expression of the propagator,

$$\begin{aligned}
U(x,t) &= \frac{1}{L}\theta(z,\tau)e^{2\pi i\left(\frac{x}{L}\lambda - \frac{\beta v t \lambda^2}{2}\right)} && \text{(S17)}\\
&\sim \frac{1}{L}\lim_{\eta\to 0^+}\sqrt{\frac{\pi}{\eta - i\pi\tau}}e^{\frac{\pi^2 z^2}{\eta - i\pi\tau}}e^{2\pi i\left(\frac{x}{L}\lambda - \frac{v t \lambda^2}{2}\right)} && \text{(S18)}\\
&\approx \frac{1}{L}\frac{1}{\sqrt{i\tau}}e^{\frac{\pi^2 z^2}{-i\pi\tau}}e^{2\pi i\left(\frac{x}{L}\lambda - \frac{v t \lambda^2}{2}\right)} && \text{(S19)}\\
&= \frac{1}{L}\sqrt{\frac{i}{vt}}e^{\frac{-i\pi\left(\frac{x}{L} - \beta v t \lambda\right)^2}{\beta v t}}e^{2\pi i\left(\frac{x}{L}\lambda - \frac{\beta v t \lambda^2}{2}\right)} && \text{(S20)}\\
&= \frac{1}{L}\sqrt{\frac{i}{vt}}e^{\frac{-i\pi}{\beta v t L^2}\left(x^2 - 2\beta v t L \lambda x + (\beta v t L \lambda)^2\right)}e^{\frac{i\pi}{\beta v t L^2}\left(2\beta v t L \lambda x - (\beta v t L \lambda)^2)\right)} && \text{(S21)}\\
&= \frac{1}{L}\sqrt{\frac{i}{vt}}e^{\frac{-i\pi}{\beta v t L^2}\left(x^2 - 4\beta v t L \lambda x + 2(\beta v t L \lambda)^2\right)} && \text{(S22)}\\
&= \frac{1}{L}\sqrt{\frac{i}{vt}}e^{\frac{-i\pi}{\beta v t L^2}\left\{(x - 2\beta v t L \lambda)^2 - 2(\beta v t L \lambda)^2\right\}} && \text{(S23)}\\
&= \beta\mathcal{N}\sqrt{\frac{i}{4\pi t}}e^{\frac{-i\beta\mathcal{N}^2 L^2}{4t}\left\{(\frac{x}{L} - 2\frac{4\pi t}{\beta\mathcal{N}^2 L^2}\lambda)^2 - 2(\frac{4\pi t}{\beta\mathcal{N}^2 L^2}\lambda)^2\right\}} && \text{(S24)}\\
&= \mathcal{A}e^{i\mathcal{B}S_{\text{periodic}}} && \text{(S25)}
\end{aligned}$$

Arriving here, we have demonstrated that the inclusion of the Bloch momenta ($\approx \lambda$ in our derivation) in the periodic system modifies the action as S_{periodic} from the free particle one S_{free} (see, e.g., [S1]), which is given as follows:

$$U_{\text{free}}(x,t) = \left(\frac{m}{2\pi i\hbar t}\right)^{\frac{1}{2}} e^{\frac{im}{2\hbar t}x^2} = \mathcal{A}'e^{i\mathcal{B}'S_{\text{free}}}$$

Because the change of the action implies the transformation of the propagating distance of the electromagnetic waves, we can equivalently realize in the single cavities the effects of the action change arising due to the Bloch momenta in the cavity lattices by deforming the boundary shape of the single cavities.

[S1] Schulman, Lawrence S. *Techniques and applications of path integration.* Courier Corporation, 2012.

S2 Effective equivalence between boundary deformation and Bloch momenta

Based on the qualitative argument given above, in this section, we explicitly show that the inclusion of the Bloch momentum in the cavity lattices corresponds to the perturbation of the additional boundary deformation in the single cavities.

S2.1 Two-band Hamiltonian of QBT for cavity lattices and its interpretation in terms of a single cavity deformation

The effective Hamiltonian in Eq. (2) that describes the Quadratic Band Touching (QBT) in the main text is explicitly written as,

$$\begin{aligned} H &= \frac{\Delta}{2}\left(\cos k_x - \cos k_y\right)\sigma_x + V_0\left(\sin k_x \sin k_y\right)\sigma_z \\ &= \begin{pmatrix} V_0 \sin k_x \sin k_y & \frac{\Delta}{2}\left[\cos k_x - \cos k_y\right] \\ \frac{\Delta}{2}\left[\cos k_x - \cos k_y\right] & -V_0 \sin k_x \sin k_y \end{pmatrix} \end{aligned} \tag{S26}$$

where Δ, V_0, and σ_i denote the mode coupling, the relative energy shift, and the Pauli matrix, respectively. Eq. (S26) has eigenvalues,

$$H_\pm = \pm\frac{1}{2}\sqrt{\Delta^2\left[\cos k_x - \cos k_y\right]^2 + V_0^2 \sin^2 k_x \sin^2 k_y} \tag{S27}$$

The first coupling term (Δ) and the second energy shift term (V_0) in the square-root in Eq. (S27) can be translated into the cavity boundary deformation as follows:

(A) When $(k_x \neq 0,\ k_y \neq 0)$ and $k_x = k_y$, the coupling term associated with Δ vanishes so that only the relative energy shift of V_0 induces the split energy gap.

  → The energy shift can be translated into the additional deformation of the boundary shape in which only one cavity direction is elongated in either $\theta = -\pi/4$ (and $-5\pi/4$) or $\pi/4$ (and $5\pi/4$) directions with the strength ε_1. These directions follow the ones that the two spinor modes are mainly localized.

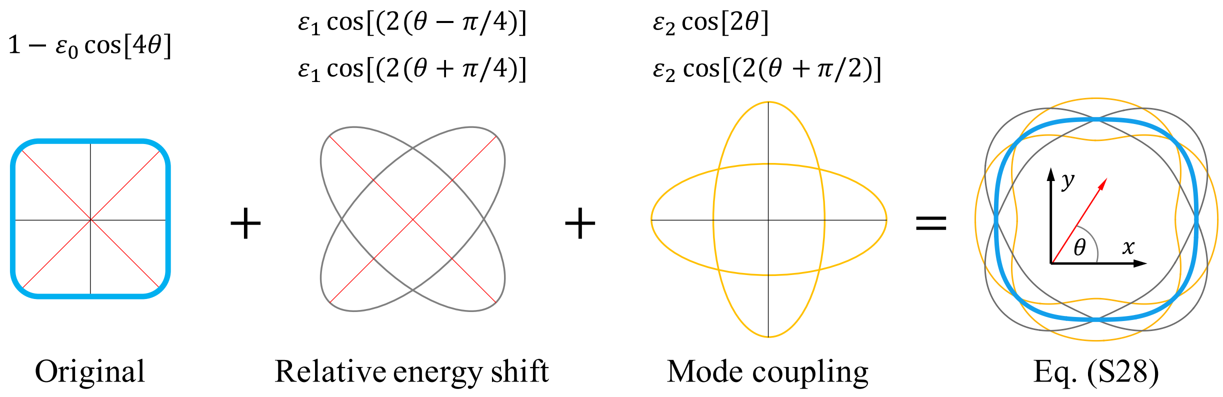

Figure S1: A newly defined single cavity boundary designed to reflect the perturbation of modes which is equivalent to the ones arising due to Bloch momentum in the lattice system. The original 4-fold symmetric cavity shape is further deformed in the directions $\theta = \pm\pi/4$ (red diagonal lines) and $(0, \pi/2)$ (black horizontal and vertical lines) in order to, respectively, take into account the relative energy shifts and the mode couplings.

(B) When $(k_x = 0,\ k_y \neq 0)$ or $(k_x \neq 0,\ k_y = 0)$, a non-zero coupling associated with Δ gives rise to the split energy gap while the relative energy shift of V_0 vanishes.

$\rightarrow$ The coupling can be realized by the additional deformation of the boundary shape that the cavity is elongated in either $\theta = 0$ (and π) or $\pi/2$ (and $3\pi/2$) directions with the strength ε_2. These directions correspond to the ones pointing to the angle between the directions where the two spinor modes are mainly localized.

(C) For arbitrary non-zero $(k_x,\ k_y)$, the effects of the coupling and the energy shift induce the energy band gap simultaneously and continuously.

S2.2 Realization of the boundary deformation in a single cavity corresponding to the Bloch momentum in cavity lattices

The above three correspondences between the Bloch momentum in the cavity lattices and the boundary deformation can be incorporated in the single cavity by taking into account the additional boundary perturbation which is smooth and continuous.

S2.2.1 Basis shape function of the boundary deformation perturbation

On the basic C_4-symmetric cavity boundary ("Original" in Fig. S1), we add-on the perturbation of the boundary elongation along the two diagonal directions ("Relative energy shift" in Fig. S1) according to (A). Then, we further include the deformation along the horizontal and the vertical direction ("Mode coupling" in Fig. S1) according to (B). Since the harmonic perturbation of $\cos[2(\theta-\theta_0)]$ gives rise to the smooth bi-directional boundary lengthening (around $\theta = \theta_0,\ \theta_0+\pi$) and shortening (around $\theta = \theta_0 \pm \pi/2$), we adopt it as a basis shape function to realize our perturbation of the directional boundary elongation.

S2.2.2 Bloch momentum direction and strength of the boundary deformation perturbation

The basis functions of the directional boundary perturbation for the single cavity, which are prepared above, should be controlled smoothly to properly emulate the Bloch momentum involved in the lattice systems.

For the ε_1-perturbation of "Relative energy shift" in Fig. S1, if the Bloch momentum points to $\phi = \tan^{-1}(k_y/k_x) = -\pi/4$, the boundary elongation along only this direction (and $\phi+\pi$) should take place, and the other one for $\phi = +\pi/4$ (and $\phi+\pi$) should disappear. This can be achieved by introducing the coefficients $\cos^2(\phi-\pi/4)$ and $\sin^2(\phi-\pi/4)$. Note that here the squared harmonic terms are considered to make them positive values in both directions of ϕ and $\phi+\pi$ gently. For the same reason, the ε_2-perturbation of "Mode coupling" in Fig. S1 should have coefficients $\cos^2\phi$ and $\sin^2\phi$. Gathering together the original boundary shape as well as all the terms of additional perturbations emulating the Bloch momentum, the single

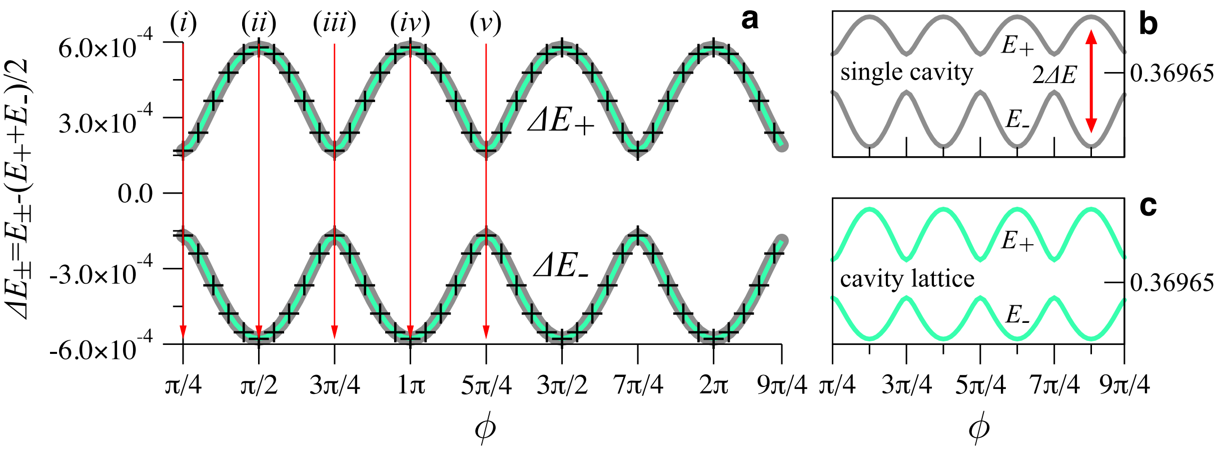

Figure S2: Comparison of the energy dispersion of the single cavity, the cavity lattice, and the model Hamiltonian. The two thick curves ($\Delta E_\pm$) in **a** are the rearranged energies (about the mean energy $E_\pm$) of the cavity lattice obtained as a function of the Bloch momentum $(k_x, k_y) = |\mathbf{k}|(\cos\phi, \sin\phi)$ in **c**, $|\mathbf{k}| = \pi/2$. The overlaid thin curves in **a** correspond to the ones of the single cavities obtained in **b** using the boundary shape given in Eq. (S28). The used parameters are $(\varepsilon_0, \varepsilon_1, \varepsilon_2) = (5\times10^{-2}, 1.33\times10^{-3}, 5.59\times10^{-3})$. Discrete "plus" symbols in **a** represent the eigenvalues Eq. (S27) with $(\Delta, V_0) = (1.156\times10^{-3}, 2.13\times10^{-4})$. For the eigenvalues, the same Bloch momentum (k_x, k_y) employed in the cavity lattice case is used. Note that (Δ, V_0) in our model Hamiltonian does not change if we consider the same energy bands. The arrows labeled by $[(i), (ii), (iii), (iv), (v)]$ mark the selected Bloch momentum and the corresponding deformation to examine wavefunctions of modes in the cavity lattice [Fig. S3] and in the single cavity [Fig. S4]. Note that, without loss of generality, the abscissa in **a**, **b**, and **c** starts by $\phi = \pi/4$ not 0 in order to see the mode evolution from the fundamental spinor states formed near $k_x = k_y$ (see Figs. S3 and S4).

cavity boundary shape yields as follows:

$$
\begin{aligned}
\frac{r(\theta, \varepsilon_0;\ \phi, \varepsilon_1, \varepsilon_2)}{r_0} &= 1 - \varepsilon_0 \cos[4\theta] \\
&\quad + \varepsilon_1 \cos[2(\theta - \pi/4)] \cos^2[\phi - \pi/4] + \varepsilon_1 \cos[2(\theta + \pi/4)] \sin^2[\phi - \pi/4] \\
&\quad + \varepsilon_2 \cos[2\theta] \cos^2[\phi] + \varepsilon_2 \cos[2(\theta + \pi/2)] \sin^2[\phi] \\
&= 1 - \varepsilon_0 \cos[4\theta] + \varepsilon_1 \sin[2\theta] \sin[2\phi] + \varepsilon_2 \cos[2\theta] \cos[2\phi] \qquad \text{(S28)}
\end{aligned}
$$

where $\theta = \tan^{-1}(y/x)$ and $\phi = \tan^{-1}(k_y/k_x)$.

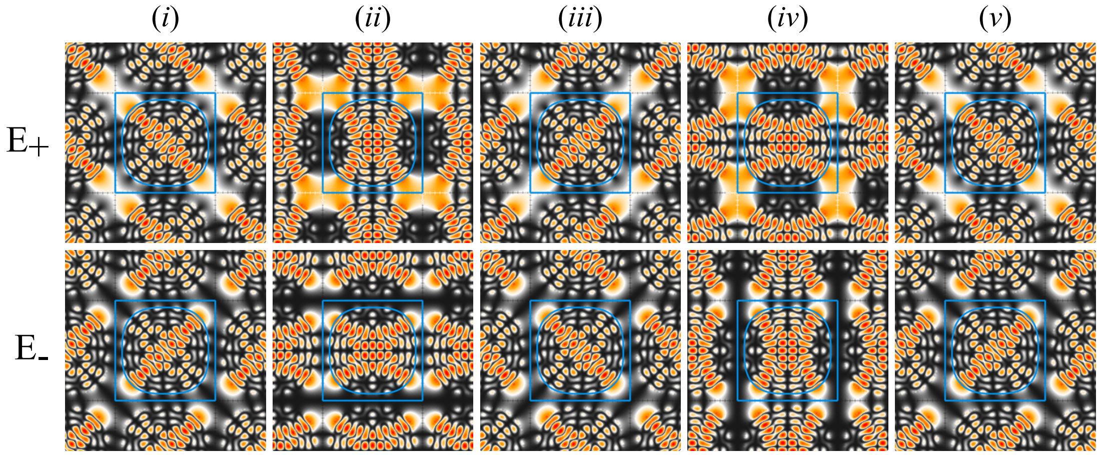

Figure S3: Cavity lattice: Selected wavefunctions $|\psi|^2$ of the modes in the cavity lattice, as a function of the continuous variation of the Bloch momentum. The above $(+)$ and below $(-)$ ones in the same columns, labeled as $[(i), (ii), (iii), (iv), (v)]$, correspond to the modes of ΔE_+ and ΔE_- in Fig. S2**a** for (k_x, k_y) marked by the same labels $[(i), (ii), (iii), (iv), (v)]$. Please find the attached movie files "Ani_supple_phc_wav1.mov" and "Ani_supple_phc_wav2.mov" for the entire mode evolution over $\pi/4 \leq \phi \leq 9\pi/4$.

S2.2.3 Equivalence of the energy dispersion due to the Bloch momenta and the boundary deformation

In Fig. S2, we compare the Bloch momentum-dependent energy of the spinor modes in the cavity lattice (correspond to Fig. 3 in the main text) with the single cavity modes perturbed according to Eq. (S28) as well as the eigenvalues in Eq. (S27) of the model Hamiltonian. In the figure, we can confirm the perfect agreement among the three computations with the parameters $(\varepsilon_0, \varepsilon_1, \varepsilon_2) = (5 \times 10^{-2}, 1.33 \times 10^{-3}, 5.59 \times 10^{-3})$ which are fixed numerically. To clearly demonstrate that the Bloch momenta in the cavity lattice can equivalently bring a strong perturbation in the single cavity, the large Bloch momenta, as $|\mathbf{k}| = \pi/2$, is examined in Fig. S2.

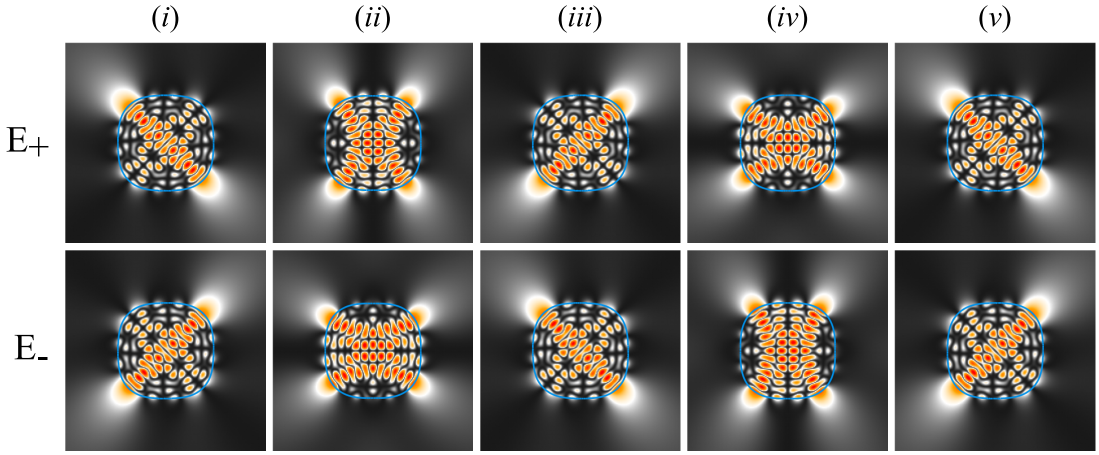

Figure S4: Single cavity: Selected wavefunctions $|\psi|^2$ of the single cavity modes, as a function of the continuous variation of ϕ in the additional deformation given in Eq. (S28). The above (+) and below (−) ones in the same columns, labeled as $[(i),(ii),(iii),(iv),(v)]$, correspond to the modes of ΔE_+ and ΔE_- in Fig. S2**a** for the parameters marked by the same labels $[(i),(ii),(iii),(iv),(v)]$. Please find the attached movie files "Ani_supple_single_wav1.mov" and "Ani_supple_single_wav2.mov" for the entire mode evolution over $\pi/4 \leq \phi \leq 9\pi/4$.

S2.2.4 Equivalence of the wavfunction evolution for the Bloch momenta and the doundary deformation

We further verify the morphological resemblance of modes evolution between the ones in the cavity lattice, given as a function of the Bloch momentum, and those computed in the single cavity, as a function of the corresponding additional deformation given in Eq. (S28). In Figs. S3 and S4, we can transparently identify that the wavefunctions obtained for the variation of the Bloch momentum in the cavity lattice are equivalent to those obtained for the additional deformation in the single cavity described by the boundary shape given in Eq. (S28).

S2.2.5 Poincaré section of phase space

So far, we have proven that we can effectively transduce the Bloch momenta in the cavity lattice into a single cavity by introducing the perturbation of additional deformations. In this section, we show that the phase space is also altered singnificantly because of this new perturbed

boundary shape.

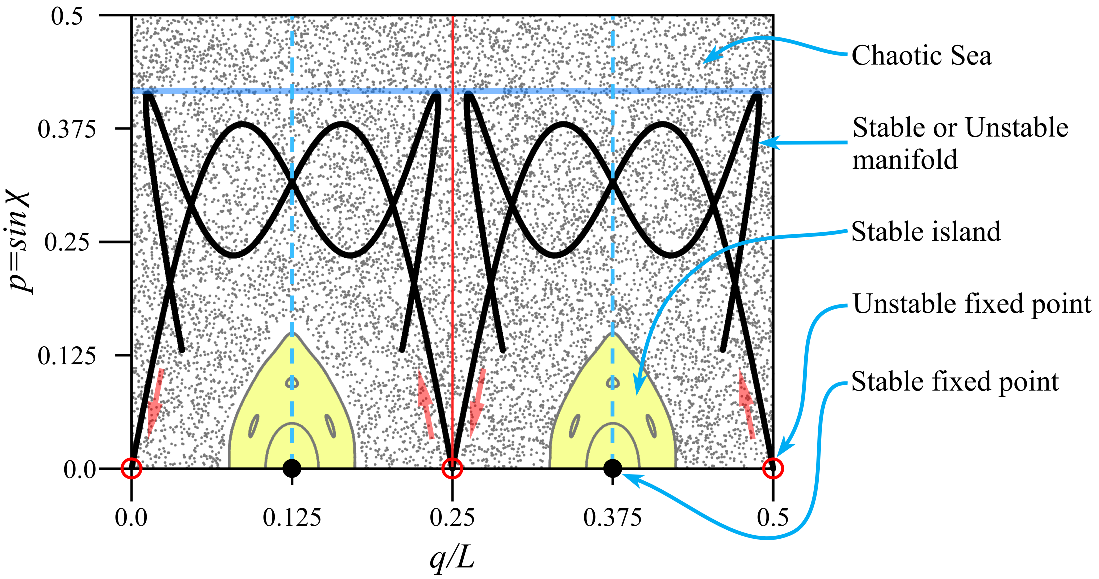

Figure S5: Phase space $(q, p) \in [0, L/2] \times [0, 0.5]$ in Birkhoff-coordinate obtained for the original cavity without the additional deformation perturbation, i.e., $(\varepsilon_0, \varepsilon_1, \varepsilon_2) = (5 \times 10^{-2}, 0, 0)$ in Eq. (S28). The phase space consists of mixed components; a chaotic sea, a stable (unstable) manifold, a stable island, an unstable fixed point, and a stable fixed point, are indicated by (blue) arrows. The outward and inward (red) arrows toward the unstable fixed points represent the unstable and stable manifold directions, respectively. The manifolds are obtained after two-bounce iterations of the ray propagation using 10^6 initial sets of $(q, p) \in [q_0 - \delta q, q_0 + \delta q] \times [-\delta p, \delta p]$, where $(\delta q, \delta p) = (5 \times 10^{-2} L, 1 \times 10^{-5})$. According to the original C_4-symmetry of the cavity boundary, the phase space exhibit symmetries as well; a translation symmetry with a period $\Delta q = L/4$ (see the vertical-solid line marking the unstable fixed point). In addition, the phase space also show the mirror symmetry with respect to the vertical-dashed lines marking the stable fixed points. The thick horizontal lines mark the equi-highest points of the manifolds to stress the symmetric phase space structures in contrast to the asymmetric ones in Fig. S6.

When the cavity is not perturbed by the additional deformation, i.e., $(\varepsilon_0, \varepsilon_1, \varepsilon_2) = (5 \times 10^{-2}, 0, 0)$ in Eq. (S28), the corresponding phase space preserves a translation symmetry with a period $\Delta q = L/4$ and a mirror symmetry about the axes in the interval $\Delta q = L/8$, where L is the full perimeter of the cavity boundary. In Fig. S5, these symmetries are clarified by examining the symmetric phase space components: the mixed phase space structures of the chaotic sea embedding the isolated unstable fixed points and the stable islands exhibit the translation and

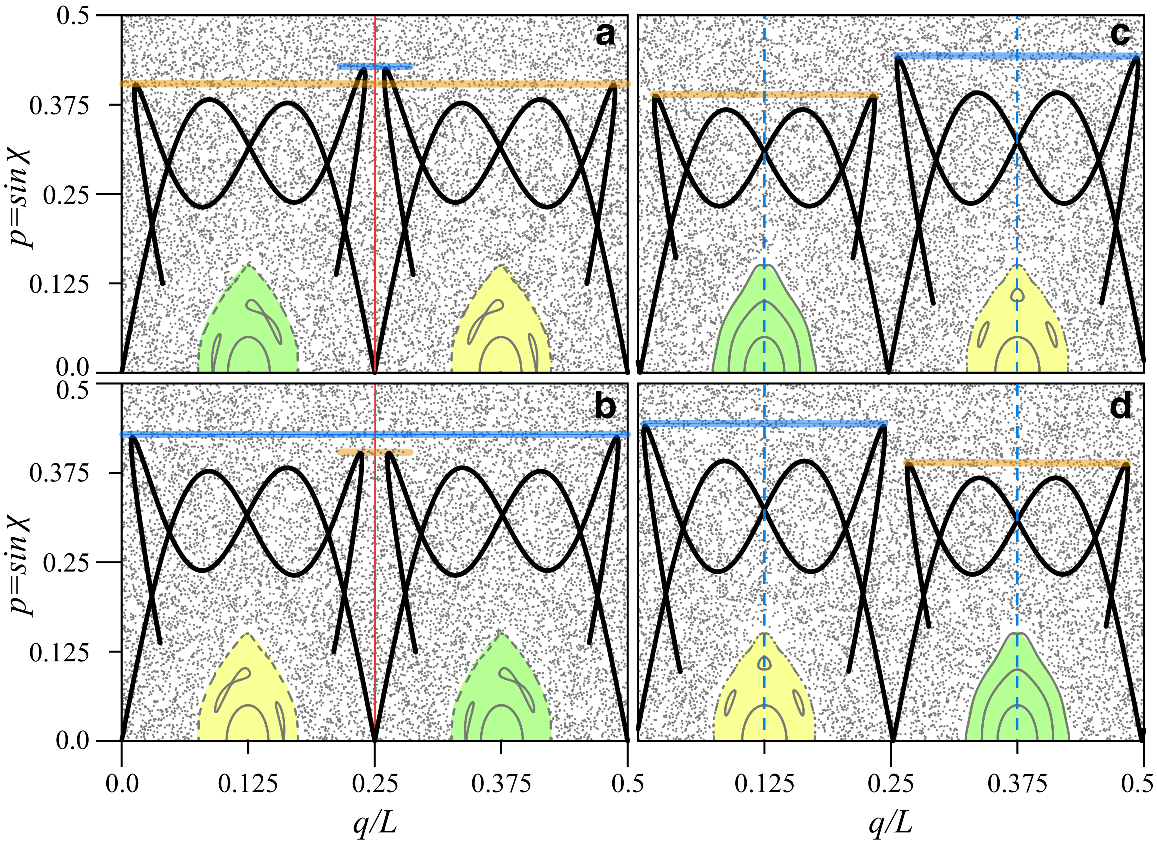

Figure S6: Phase space in $(q, p) \in [0, L/2] \times [0, 0.5]$ obtained for the perturbed cavity by Eq. (S28) with $(\varepsilon_0, \varepsilon_1, \varepsilon_2) = (5 \times 10^{-2}, 1.33 \times 10^{-3}, 5.59 \times 10^{-3})$. **a** and **b** are for $\phi = \pm\pi/4$ corresponding to (i) in Fig. S2**a**. **c** and **d** are for $\phi = \pm\pi/2$ corresponding to (ii) in Fig. S2**a**. In **a**-**d**, the stable and unstable manifolds (thick dispersive curves) are overlaid on the chaotic sea (underlying gray dots). The vertical-solid (dashed) lines in **a** and **b** (**c** and **d**) stand for the mirror symmetry axis of the phase space. The thick horizontal lines in **a**-**d** mark the pairwise equi-highest points of the manifolds to prove the asymmetric phase space compared to the symmetric ones in Fig. S5. Also, the morphologically different island structures are shaded by different colors. The phase space evolution over the full range of angle $\phi \in [\pi/4, 9\pi/4]$ can be found in the supplementary animation: "Ani_supple_sos_manifold.mov".

mirror symmetries. According to Liouville's theorem for the Hamiltonian chaos, the unstable fixed point is the hyperbolic one governing the stable and unstable manifold, i.e., the phase space volume is preserved. In demonstrations, the manifolds are obtained after two-bounce iterations of the ray propagation using 10^6 initial sets of $(q, p) \in [q_0 - \delta q, q_0 + \delta q] \times [-\delta p, \delta p]$,

where $(\delta q, \delta p) = (5 \times 10^{-2} L, 1 \times 10^{-5})$, embedding the four unstable fixed points of the bouncing ball orbit; $\{(q_0, p): (0,0), (L/4, 0), (2L/4, 0), (3L/4, 0)\}$ and excluding the island region. At the center of the islands, the stable fixed points are located.

Now, turning on the additional deformation perturbation of $(\varepsilon_1, \varepsilon_2)$ breaks C_4-symmetry of the cavity boundary shape and reduces it to C_2. As a consequence, the symmetry of the corresponding phase space is altered as well. Figure S6 shows the phase space of the perturbed single cavities described by Eq. (S28) with $(\varepsilon_0, \varepsilon_1, \varepsilon_2) = (5 \times 10^{-2}, 1.33 \times 10^{-3}, 5.59 \times 10^{-3})$: **a** and **b** with $\phi = \pm\pi/4$, i.e., (i) in Fig. S2**a**; **c** and **d** with $\phi = \pm\pi/2$, i.e., (ii) in Fig. S2**a**. We can see in the figures that the $L/4$ translation symmetry is broken in all cases **a**-**d**, which was preserved in Fig. S5 obtained for the C_4-symmetric cavity. Only the mirror symmetry resulting from the reduced C_2-symmetry of the cavity boundary is shown: about the vertical-solid line in **a** and **b**; about the vertical-dashed line in **c** and **d**. The pairwise equi-level highest points of the manifold structures (thick dispersive curves) are marked by the thick horizontal lines to prove the asymmetric phase space. The broken translation symmetry is confirmed again by the different color-shaded islands.

In conclusion, we have demonstrated that the Bloch momentum in the cavity lattices can be equivalently transduced into the perturbation of the boundary deformation in the single cavity system. We emphasize that the boundary distortion brought by the additional deformation perturbation is strong enough to significantly reconfigure the phase space, as is clarified in Fig. S6. We further stress that the distortion of the phase space is, in fact, much more crucial and complicated if all the detailed structures of the islands and the manifolds are examined, though here we only exemplify the simple ones (but evident) for convenient comparison.

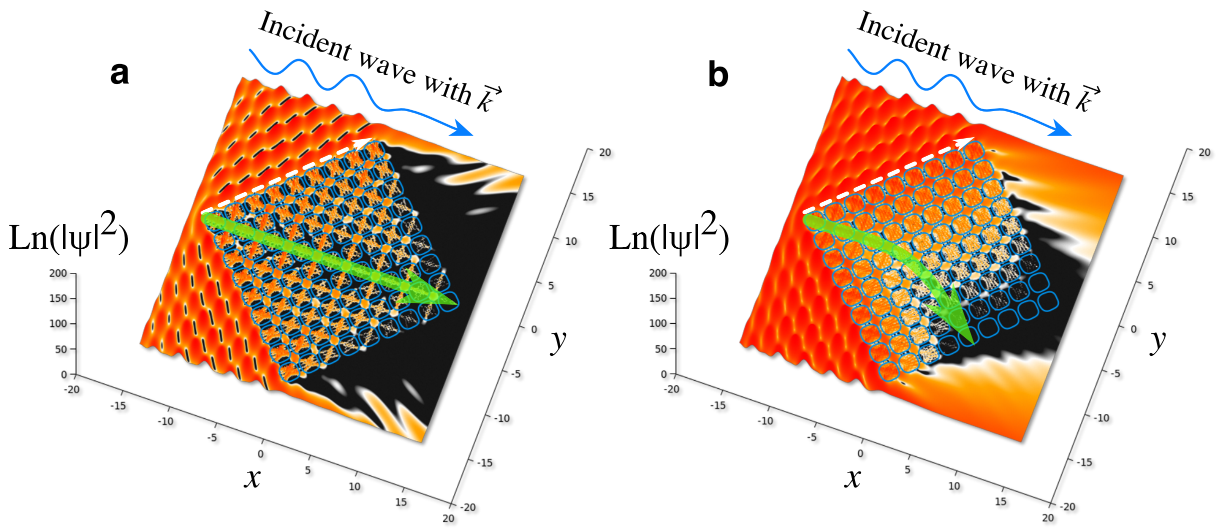

Figure S7: Light scattering of an incident wave impinging on the finite-sized cavity array (9×9) in the direction $\vec{k} = (k_x, 0)$. **a** Without the Berry curvature $\Omega = 0$: the boundary shape of the cavities is 4-fold symmetric; $r_0(\theta) = R_0[1 - \varepsilon_0 \cos(4\theta)]$, $\varepsilon_0 = 0.05$. Along the arrowed-dashed line the refractive index is varied as $[10 - \delta n, 10 + \delta n]$, $\delta n \sim 1 \times 10^{-4}$, to realize $\dot{\vec{k}}$ in Eq. (4) in the main text. The trivial light transportation takes place (straight thick arrowed line). **b** With the Berry curvature $\Omega \neq 0$: the boundary shape is further deformed as $r(\theta) = r_0(\theta) - \varepsilon_p \cos[N\theta + \phi_p]$, $(N, \varepsilon_p, \phi_p) = (5, 0.01, \pi/10)$. The skew light transportation takes place (curved thick arrowed line) due to the non-zero term of $\dot{\vec{k}} \times \Omega_{xy}(\vec{k})\hat{z}$ in Eq. (4) in the main text.

S3 Skew scattering light transportation

In the main text, we discuss the skew light transportation arising due to the non-zero finite value of the Berry curvature when the C_4-symmetry of the cavity boundary is broken. The skew light transportation is governed by Eq. (4) in the main text. Here, we explicitly demonstrate this skew light transportation by exemplifying the finite-size (9×9) cavity arrays. By comparing the trivial light transportation in the array of the cavity having a C_4-symmetric boundary [no Berry curvature; Fig. S7**a**] with the one with the cavity with a broken C_4-symmetric boundary [with Berry curvature; Fig. S7**b**], we can confirm that the skew light transportation can, indeed, be induced by the Berry curvature.
